# Gene's hubs in retinal diseases: A retinal disease network

**DOI:** 10.1016/j.heliyon.2018.e00867

**Published:** 2018-11-02

**Authors:** J.M. Lázaro-Guevara, B.J. Flores-Robles, K. Garrido, V. Pinillos-Aransay, A. Elena-Ibáñez, L. Merino-Meléndez, J.A. López-Martínez, R. Victoriano-Lacalle

**Affiliations:** a Department of Human Genetics, University of Utah, Salt Lake City, Utah, USA; b Rheumatology Department, San Pedro Hospital, Logroño, Spain; c Paediatrics Department Guatemalan Social Secure Guatemala, Guatemala; d Family Medicine, Nájera, Logroño, Spain

**Keywords:** Genetics

## Abstract

**Background:**

Retinal diseases associated with the dysfunction or death of photoreceptors are a major cause of blindness around the world, improvements in genetics tools, like next generation sequencing (NGS) allows the discovery of genes and genetic changes that lead to many of those retinal diseases. Though, there very few databases that explores a wide spectrum of retinal diseases, phenotypes, genes, and proteins, thus creating the need for a more comprehensive database, that groups all these parameters.

**Methods:**

Multiple open access databases were compiled into a new comprehensive database. A biological network was then crated, and organized using Cytoscape. The network was scrutinized for presence of hubs, measuring the concentration of grouped nodes. Finally, a trace back analysis was performed in areas were the power law reports a high r-squared value near one, that indicates high nodes density.

**Results:**

This work leads to creation of a retinal database that includes 324 diseases, 803 genes, 463 phenotypes, and 2461 proteins. Four biological networks (1) a disease and gene network connected by common phenotypes, (2) a disease and phenotype network connected by common genes, (3) a disease and gene network with shared disease or gene as the cause of an edge, and (4) a protein and disease network. The resulting networks will allow users to have easier searching for retinal diseases, phenotypes, genes, and proteins and their interrelationships.

**Conclusions:**

These networks have a broader range of information than previously available ones, helping clinicians in the comprehension of this complex group of diseases.

## 1 Introduction

Retinal diseases affect 1 in 1,200 people throughout the world [1]. For example, one disease, retinitis pigmentosa (RP), is a class of inherited degenerative eye diseases caused by genetic mutations. It is possible that these diseases result from several different mutations and share molecular features, given that many molecular components of the human cell are dependent on one another. Network medicine is an approach that aims to understand the molecular complexity of specific diseases and the molecular relationships among different diseases. The many diseases that are classified as RP are likely to have molecular relationships [2, 3, 4, 5].

RP affects 1 in 3,500 people in the United States and Europe [4]. This class of diseases is characterized by mutations in the genes that produce the photoreceptors or the retinal pigment epithelium of the retina leading to visual impairment and eventual blindness, severely impacting the quality of life of the patients affected. Retinitis pigmentosa is exceptionally heterogeneous. This includes genetic heterogeneity (many different genes may cause the same disease phenotype); allelic heterogeneity (there may be many different disease-causing mutations in each gene); phenotypic heterogeneity (different mutations in the same gene may cause different diseases); and clinical heterogeneity (the same mutation in different individuals may produce different clinical consequences, even among members of the same family). The extent of heterogeneity of RP and most retinal diseases can be confusing to patients and clinicians alike and is a confounding factor in diagnosis. Therefore, understanding the molecular relationships between these diseases will allow us to discover the biological significance of genetic mutations causing diseases and to identify drug targets and biomarkers to aid in finding an eventual cure [5, 6].

However, actual databases are incomplete or networks lack on all possibly related information. The hypothesis of this research is that if retinal diseases, phenotypes, genes, and proteins are put into one database, the interactions can be studied and mapped into a network. If the biological network is created, then the nodes that have significantly greater number of associations (**hubs**) in comparison to others can be identified and analyzed [7].

When addressing this problem there are a couple main challenges. First, compiling a complete relational database including retinal diseases, phenotypes, genes, and proteins is challenging. This challenge includes quarrying through several previously existing databases that include parts of the needed information and compiling that information. Another main challenge is visualizing the complex database in a network that portrays the relevant information and patterns.

Once a more complete retinal network including diseases, phenotypes, genes, and proteins is created, the benefits are great. Firstly, the knowledge of hubs in the network can focus future research and medicine efforts on the parts of the network that have the greatest effect. Additionally, a completed retinal network can guide clinicians to improve patient specific treatment. The presence of a network and database that includes a wide variety of retinal diseases will also increase the coverage of this solution [8].

The current state-of-the-art method for looking at relationships between retinal diseases, phenotypes, genes, and proteins is to search through the various databases and find the relative information. This method is slow and makes it very easy to obtain miss desired information. This method also does not allow visualization of the relationships present.

Another method includes the use of the RPGeNet (https://compgen.bio.ub.edu/RPGeNet/). RPGeNet is a good initial retinitis pigmentosa gene network, but it does not cover all the properties that may be of interest to researchers and physicians. Additionally, RPGeNet only covers information regarding retinitis pigmentosa, and does not contain information of other retinal diseases [1].

The approach used in this paper will start by gathering a detailed set of information of many different diseases, phenotypes, genes, and proteins from a variety of sources. This will improve on the variety and coverage of information included in the dataset.

Disease networks have been made before, but never for retinal diseases as a whole. One example of a currently existing disease network is a network containing 727 diseases grouped by disorder class [1, 3, 8, 9].

## 2 Methods

The methods include details of the databases used in this paper, methods for database and network creation, and assessment protocol. In overview, all the process started by identifying databases that contained the information needed to collect in the database. Then, those databases were mined for the desired information using SQL queries and Python scripts. The information was filtered and packed in Microsoft Access, for posterior relationship creation.

### 2.1 Databases

In order to create a database that includes retinal diseases, phenotypes, genes, and proteins all the information was retrieved from a variety of datasets and databases. The description of each dataset and database used is described here.

A variety of datasets was used to allow a more complete database to be assembled. The biggest dataset used was RetNet, which is a retinal information network. This dataset is availible at https://sph.uth.edu/Retnet/. RetNet contains genes and mapped loci causing retinal diseases [8].

RetinoGenetics dataset. This dataset can be reached at http://www.retinogenetics.org/Analysis/ana2/ [10]. The RetinoGenetics dataset contains retinal diseases, gene symbols, gene location, and corresponding OMIM ID.

The OMIM database was also incorporated. This database can be found at https://www.omim.org/. The OMIM database was used to find relationships between genes, gene locations, phenotypes, and diseases [11, 12].

The UniProt database was used. This database is available at http://www.uniprot.org/mapping/. The UniProt database was used for information regarding proteins related to the disease genes [13, 14].

The DisGeNET database was used to retrieve information of retinal disease gene ID's and names. This database can be found at http://www.disgenet.org/web/DisGeNET/menu [6, 15].

The Monarch Initiative database was used to retrieve retinitis pigmentosa and related diseases' genes, phenotypes, genotypes, models, and variants. This database can be found at https://monarchinitiative.org/disease/DOID:10584 [16].

### 2.2 Database and network creation

The retrieval process involved creating SQL queries and Python scripts to run through each database and record the desired information into compacted new Microsoft Access Database.

**Fig. 1:**
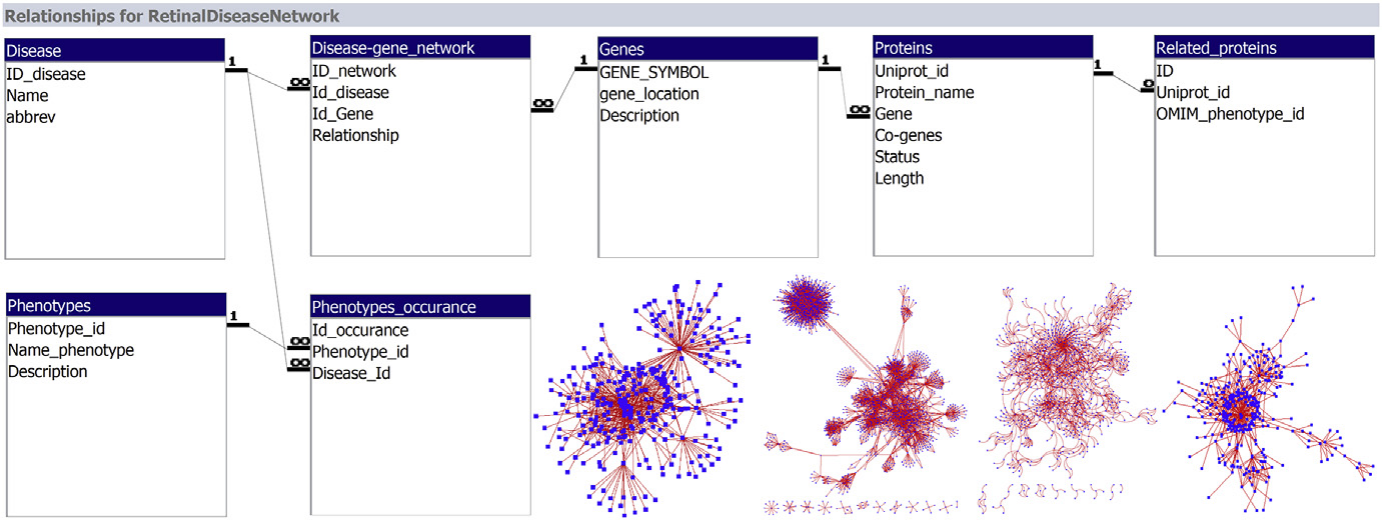
Microsoft Access relationship view of created Retinal Network Database. This figure shows the different tables that make up the created retinal database, including Disease, Disease-Gene Network, Phenotype Occurrence, Phenotypes, Genes, Proteins, and Related Proteins. The relationships are one-to-many and many-to-one. The links between tables are through ID keys, whether it be disease, gene, phenotype, or protein ID.

To set up this new database, was necessary the creation of new tables for Disease, Disease-Gene Network, Phenotype Occurrence, Phenotypes, Genes, Proteins, and Related Proteins. The layout of the Microsoft Access tables and relationships between tables can be seen in Fig. 1.

By creating grouped queries in Microsoft access, was possible to export the data directly into Cytoscape 3.6.0.

Cytoscape is a graph theory open-source software for the visual exploration of biological networks involving genes, proteins, phenotypes and other types of interactions. It offers researchers an interactive visualization interface for exploring biological pathways and interconnections, thereby facilitating the construction of interaction pathways, in these cases the hub identifications [17, 18].

This software can read a comma-separated file (csv) with nodes and edges (connections between the nodes) defined and port them into topological network visualization. Where a layout can be applied to provide a visual topological structure (Hierarchic layout), we use the layout customized option from yWorks for Cytoscape. The Hierarchical layout algorithm portraits the precedence relation of directed graphs and highlight the main direction or flow within a directed graph, the cyclic dependencies of nodes will be automatically detected and removed. Nodes will be placed in hierarchically arranged layers, the ordering of nodes within each layer is chosen in such a way that the number of edge crossings is the smallest.

Once the data was imported into Cytoscape and the most appropriated network representation was selected, we weight each one of the variables in tables to obtain a coherent data relation and visualization of interrelations on the networks, like peripheral nodes (low node to node connectivity), hubs (nodes with higher node connectivity) and superhubs (nodes that link hubs) was determined using the NetworkAnalyzer plugin developed by Assenov et al. [5, 19, 20, 21].

**Fig. 2:**
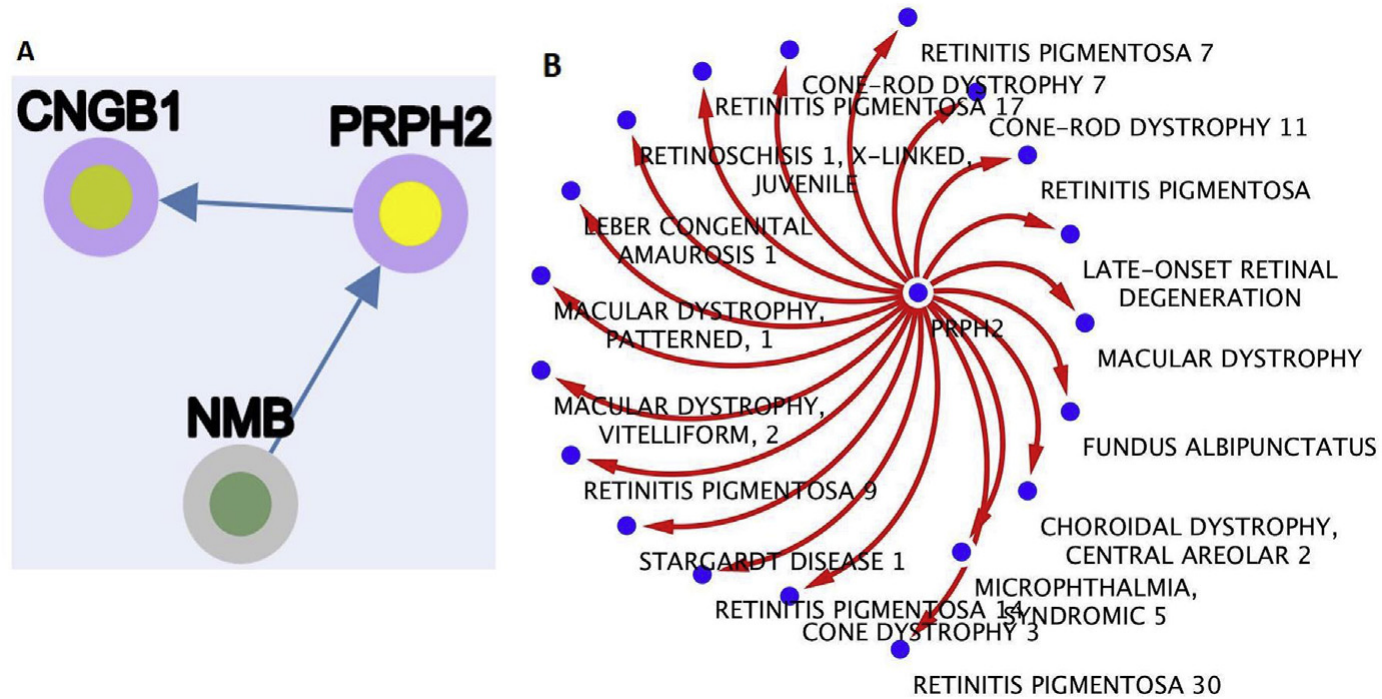
Comparison of (A) previously existing RPGeNet network to (B) newly created network to for PRPH2 gene. There is a great difference in number of connections for the PRPH2 gene depending on which network is used. This shows that this new network provides much more information that can be considered for whatever the users specifically need. This is useful for extending retinal disease research to broader areas.

The tables were converted into topological networks to show the presence or lack of hubs. Multiple potential primary hub was identified, we describe one (PRPH2) as example. This gene provides instructions for making a protein called peripherin 2. It shows several interconnected nodes, suggesting this hub importance in multiple pathways (Fig. 2).

Four networks were created from the database [1]: a disease and gene network connected by common phenotypes [2], a disease and phenotype network connected by common genes [3], a disease and gene network with shared disease or gene as the cause of an edge, and [4] a protein and disease network.

Those networks sustain as central player the disease-gen association and diverge from those players to multiple branches (e.g. genes-phenotype association). For that reason, the same player (e.g. PRPH2) can be tracked down to multiple pathways, having different node interconnectivity.

### 2.3 Assesment protocol

To analyze the networks, first each one was selected and scrutinized for presence of hubs, measuring the concentration of grouped nodes. Multiple configuration and interrelation were analyzed thought the database by looking at different disease, gene, phenotype, and protein frequency of occurrence.

After applying the network analyzer to sieve through each network, assuring the presence of hubs, using graph theory (Grafos theory). A trace back analysis was performed in areas were the model of node degrees with the power law reports a high r-squared value near one, which means hubs were present.

**Fig. 3:**
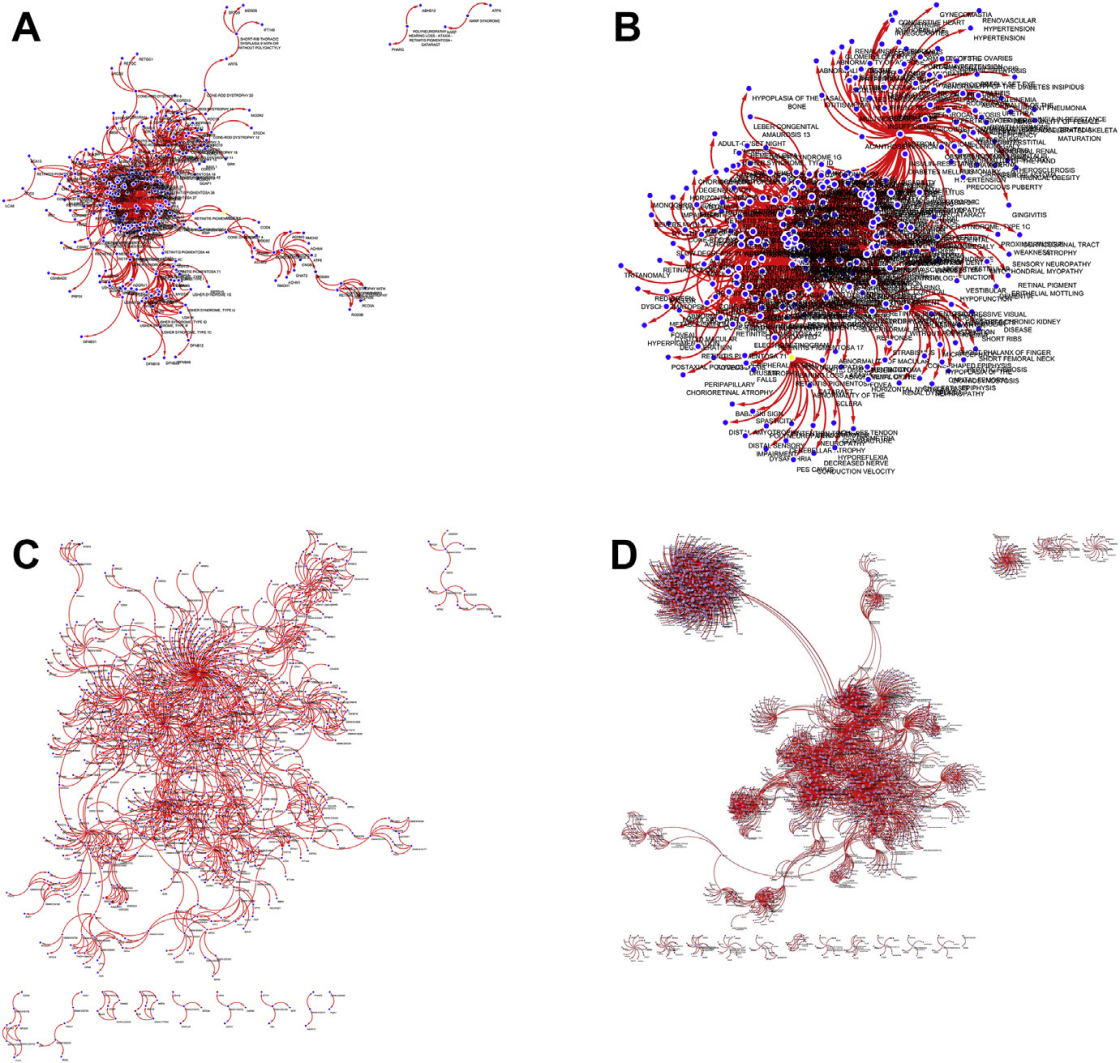
Cytoscape view of retinal networks for (A) disease-gene with common phenotypes, (B) disease-phenotype with common genes, (C) disease-gene, and (D) protein-disease. The sizes or shapes or the presence of hubs can be seen to vary between networks. Fig. 14A shows one major hub with everything extending from that. 14B shows two main hubs with smaller hubs around those. 14C shows a nice variance of node degree. 14D shows one major network with one somewhat separated hub with a network within itself.

**Fig. 4:**
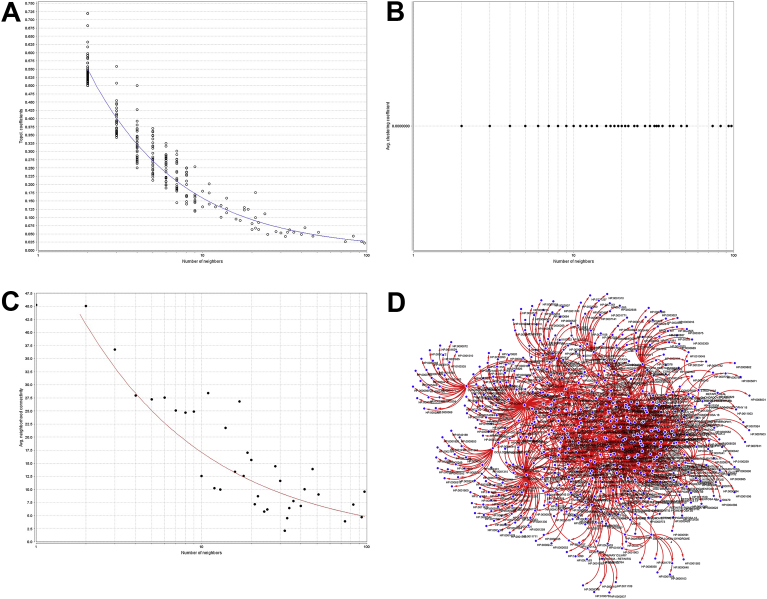
Topological Coefficients Scatterplot of Phenotype-Disease network. B) Avg. Clustering Coefficient Distribution C) Neighborhood Connectivity Distribution D) Graphical-Topological representation of subset Phenotype-Disease network. The power law model appears to be an even more accurate model showing a strong presence of hubs and a scale-free network.

The topological distribution of the networks was taken on consideration and defined as disassortative or assortative networks. The disassortative networks are spread by the repulsion of hubs (Fig. 3B and D), suggestive of a picture of modularity with nodes organized around dispersed hubs. The assortative networks (Figs. 3A, C and 4D), on the contrary, are integrated by fully connected hubs.

Those differences between topologies provides a graphical reference on the presence of clustering nodes helping in hubs identification as shown in Fig. 3.

In the topological analysis of very large networks like this. The measurement of local parameters (node vicinities) is significantly faster than the computation of global (path-related) parameters like Betweenness and closeness centralities. This increase in speed does not result on lack of power detection but needs other measurements to determine the relationship between closer nodes. It is the when measurement like Topological Coefficient overcome those limitations.

The topological coefficient (**Tn**) was calculated as described by Assenov (Centiserver plugin). The **Tn** of a node (n) with (**kn**) neighbors is the number of neighbors shared between a pair of nodes, **n** and **m**, plus one if there is a direct link between them, divided by the number of neighbors of node. Jet (**J**) of (n,m) is defined for all nodes **m** that share at least one neighbor with **n**. Therefore, **Tn** can be interpreted as the relative measure for the extent to which a node shares neighbors with other nodes [22, 23].

$$T_{n}=\frac{Avg\left(J\left(n,m\right)\right)}{K_{n}}$$

This simplistic interrelation between average (**Avg**) of local parameters and its neighbors (**k**), allows efficient hubs detection (grouped nodes interconnection), since nodes that have less than two neighbors are assigned a topological coefficient of zero, and those nodes with multiple connections are easily observed as higher Tn.

Once the hubs were localized and topological mapped, each one of this was fit in the power of law and the number of neighbors was also measured. Being marked as hubs of interest, those were no previous relation to diseases were described, or hubs that shown multiple connections to pathologies that were clinically dissimilar [7, 24, 25].

### 2.4 Using topological coefficient **(Tn)** for nodes discrimination in conjunction with centrality measures

Once the Tn is calculated, to identify relevant nodes on the biological network, protocols of analysis integrating centralities measures like radiality will improve node discrimination.

Radiality of a node **(v)** is defined as a centrality index and is calculated by computing the shortest path between the node v and all other nodes in the graph. The radiality value should be considered as an “average tendency to node concentration or isolation”, not definitively informative on the centrality of the individual node, to determine the importance of radiality on the network, this measure should be combined with other discrimination measure (e.g Tn, betweenness) [7, 24, 25].

$$C_{rad}\left(v\right)=\frac{\sum \text{w}\in \text{N}\left(\Delta \text{G}+1-\text{dist}\left(\text{v},\text{w}\right)\right)}{\text{n}-1}$$

As described before, radiality can be combined with betweenness to discriminate the presence of nodes, and the betweenness of a node **n** is calculated considering couples of nodes (v1, v2) and counting the number of shortest paths that linked them passing through a that node. Thus, a node can be traversed by only one path linking v1 and v2, but if this path is the only connecting v1 and v2 the node **n** will score a higher betweenness value. Therefore, a high betweenness score suggest that the node **n** is crucial to maintain node connections for the paths that cross them [21, 23, 26].

$$C_{spb}\left(v\right)=\sum \frac{\sum \sigma st\left(v\right)}{\sigma st}$$

For compute the centrality measures in conjunction with **Tn**, in addition to CentiServer plugin (Assenov), the use of CentiScaPe 2.2 plugin (Scardoni Group) for calculating centrality measures was implemented. The differences between centrality measures discrimination in relation to Topological Coefficient were studied by plotting centralities (Radiality and betweenness) against Tn, and later confirming the importance of nodes on a disaggregation experiment. The disaggregation experiment consist on evaluate the importance of the highlighted node by the intersection plots (eg. Tn-Radiality, Betweenness-Radiality) and evaluate the topology of the network after subtracting the highlighted node n, the dispersion and the disaggregation of the network, can be evaluated by graphical disassociation (loosing number of interconnected nodes) and taking in consideration the diameter of the network (ΔG) and a diminishing in the centroid value of the network. Since, the centroid value of a network suggests that a specific node has a central position within a graph region characterized by a high density of interacting nodes, when subtracting a node of biological importance the whole average centroid value of the network will diminish [20, 25, 26, 27, 28]. Based on the new topology both extremes of the network with Tn closer to 0 and 1 were selected to illustrate, the efficacy on node detection in conjunction with radiality as primary discriminator.

## 3 Results

This work results in the first retinal database and relational network that includes diseases, phenotypes, genes, and proteins was successfully created. The database compiled contains 324 diseases, 803 genes, 463 phenotypes, and 2461 proteins. Also, four relational trees including [1]: a disease and gene network connected by common phenotypes [2], a disease and phenotype network connected by common genes [3], a disease and gene network with shared disease or gene as the cause of an edge, and [4] a protein and disease network. The four networks can be seen in Fig. 3, and can be retrieved in Microsoft access format (.accdb), cytoscape (.cys) and interactive cytoscape.js html (.zip) at https://github.com/megahitokiri/Gene-s-hubs-in-Retinal-Diseases.

Relationship between diseases were stated by nodes comparison, as example in Table 1, OMIM# 607236 and 234200 are completely unrelated disease that share multiple genes and some diseases, OMIM# 264800 and 177850 are variant of the same disease.

**Table 1:** List comparison of two sets of diseases in close network proximity.

Figure	ID_disease	Name	Abbrev
A	OMIM:607236	HYPOPREBETALIPOPROTEINEMIA	HARP syndrome
	OMIM:234200	NEURODEGENERATION WITH BRAIN IRON ACCUMULATION 1	Hallervorden-Spatz syndrome
B	OMIM:264800	PSEUDOXANTHOMA ELASTICUM	PXE
	OMIM:177850	PSEUDOXANTHOMA ELASTICUM, FORME FRUSTRE	

**Fig. 5:**
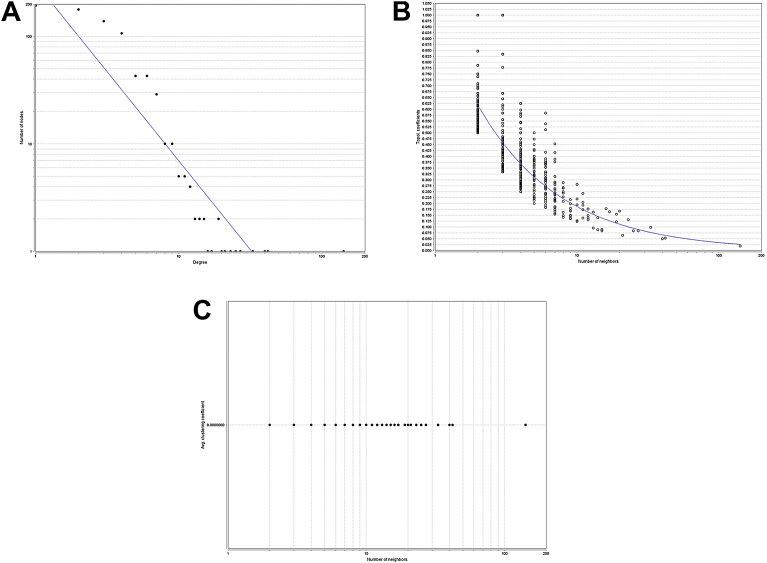
A) Node degree distribution of disease-gene network. B) Topological Coefficients scatterplot. C) Avg. Clustering Coefficient Distribution.

Each network was analyzed based on node degree distribution. The disease-gene network power law model values adequate to y = axˆb with a = 317.33, b = −1.655, correlation = 0.814, r-squared = 0.810 (on logarithmic values), which can be seen in Fig. 5A. The topological coefficient (TP) plot is shown in Fig. 5B where the power of law fits into the distribution (shown in blue line), where only one extreme value of the neighbors is over the 100 range, also visible in Fig. 5C.

**Fig. 6:**
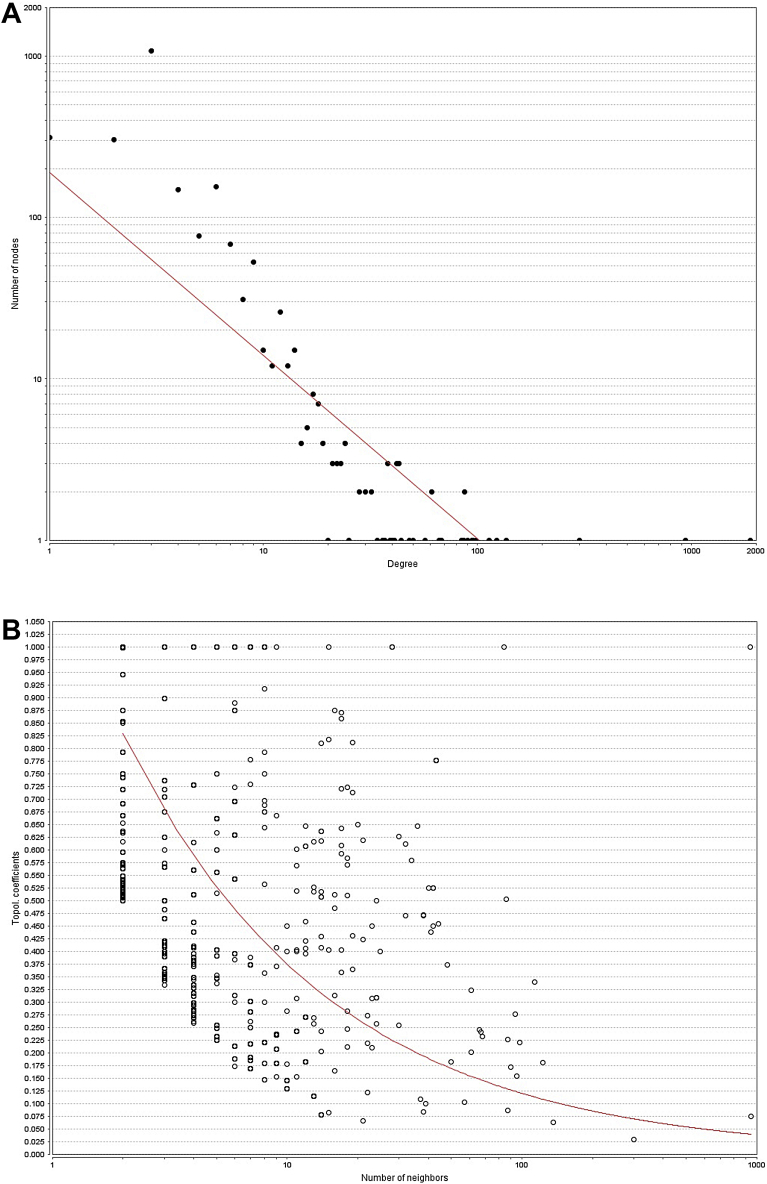
A) Node degree distribution of protein-disease network. B) Topological Coefficients Scatterplot. The power law model appears to be a moderately accurate model showing a presence of hubs. This lower correlation is most likely thrown off by outliers.

For the protein-disease network statistical analysis shows the node degree distribution power law with y = axˆb and a = 168.98, b = −1.116, correlation = 0.556, r-squared = 0.654. The model can be seen in Fig. 6A, the plot of nodes in the TP shows a high dispersion and power of law does not fits (red line) Fig. 6B.

**Fig. 7:**
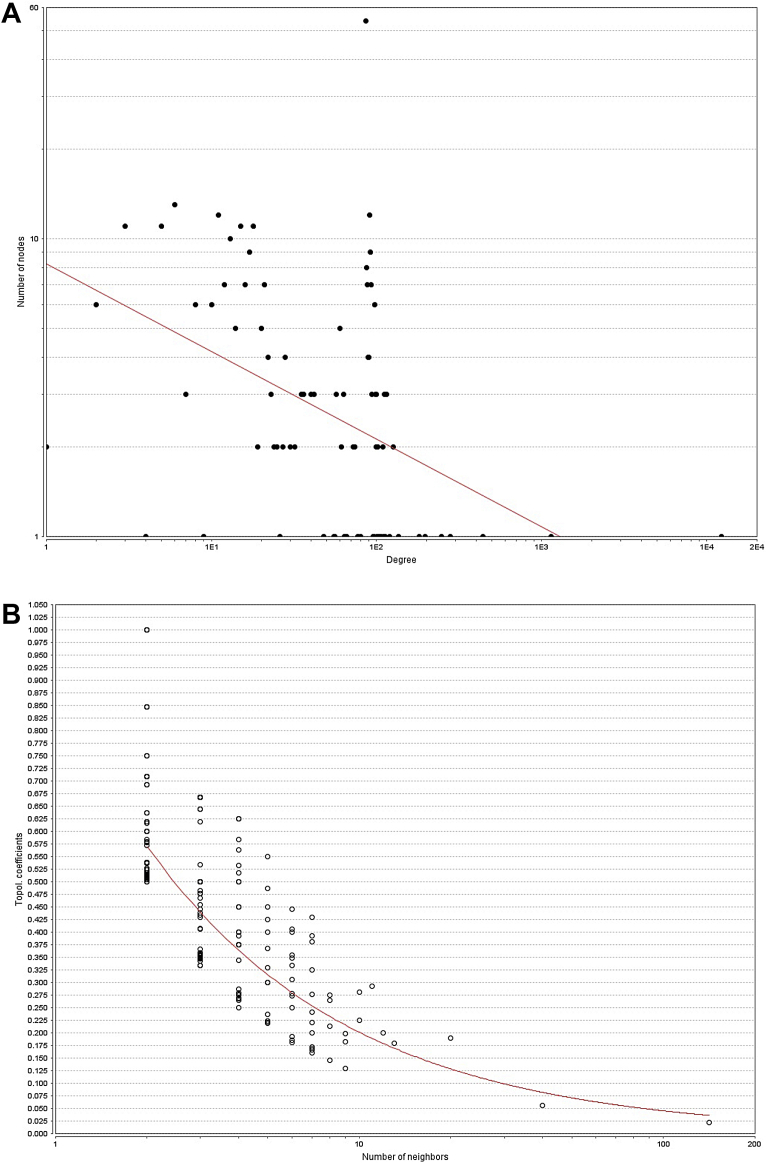
A) Node degree distribution of disease-gene by phenotype network. B) Topological Coefficients Scatterplot. The power law model does not have a high correlation showing a lack of scale-free networking. However, there are one or two hubs shown with the data points with high node degrees.

Node degree distributions for disease-gene with common phenotype the model results were y = axˆb with a = 8.245, b = −0.285, correlation = 0.171, r-squared = 0.193. Fig. 7A shows the node degree distribution. Despite the poor correlation on node degree distribution, the TP model show the presence of clustered nodes with Topological Coefficients that relays under the fitted curve (red line) Fig. 7B.

The distribution values for disease phenotype network (a subset of disease and phenotype network filtered by genes, Fig. 4D) were y = axˆb with a = 120.91, b = −1.312, correlation = 0.990, r-squared = 0.788, showing a high fit in distribution (blue line), with presence of highly concentrated nodes (Fig. 4A), and many of those with a high number of neighbors, superior to ten (Fig. 4B). That distribution makes imperative the analysis of average neighborhood per number of neighbors, showing a correlation of 0.877 and a clustering between 10 to 51 neighbors with and average inter connectivity of nodes to 14.429 (Fig. 4C).

After the analysis of phenotypes show a high presence of hubs, a table for the common phenotypic expressions found in was created (Table 2), it includes the number of appearances, and the ontology phenotype id. Being Rod-cone dystrophy the most prevalent phenotype in diseases.

**Table 2:** Ten most common phenotypes ocurring in diseases.

No.	Phenotype id	Name of phenotype	# Occurrences
1	HP:0000510	ROD-CONE DYSTROPHY	99
2	HP:0000662	NYCTALOPIA	54
3	HP:0000505	VISUAL IMPAIRMENT	48
4	HP:0000548	CONE/CONE-ROD DYSTROPHY	30
5	HP:0000613	PHOTOPHOBIA	28
6	HP:0000543	OPTIC DISC PALLOR	24
7	HP:0001133	CONSTRICTION OF PERIPHERAL VISUAL FIELD	23
8	HP:0007843	ATTENUATION OF RETINAL BLOOD VESSELS	21
9	HP:0007663	REDUCED VISUAL ACUITY	21
10	HP:0000639	NYSTAGMUS	20

**Fig. 8:**
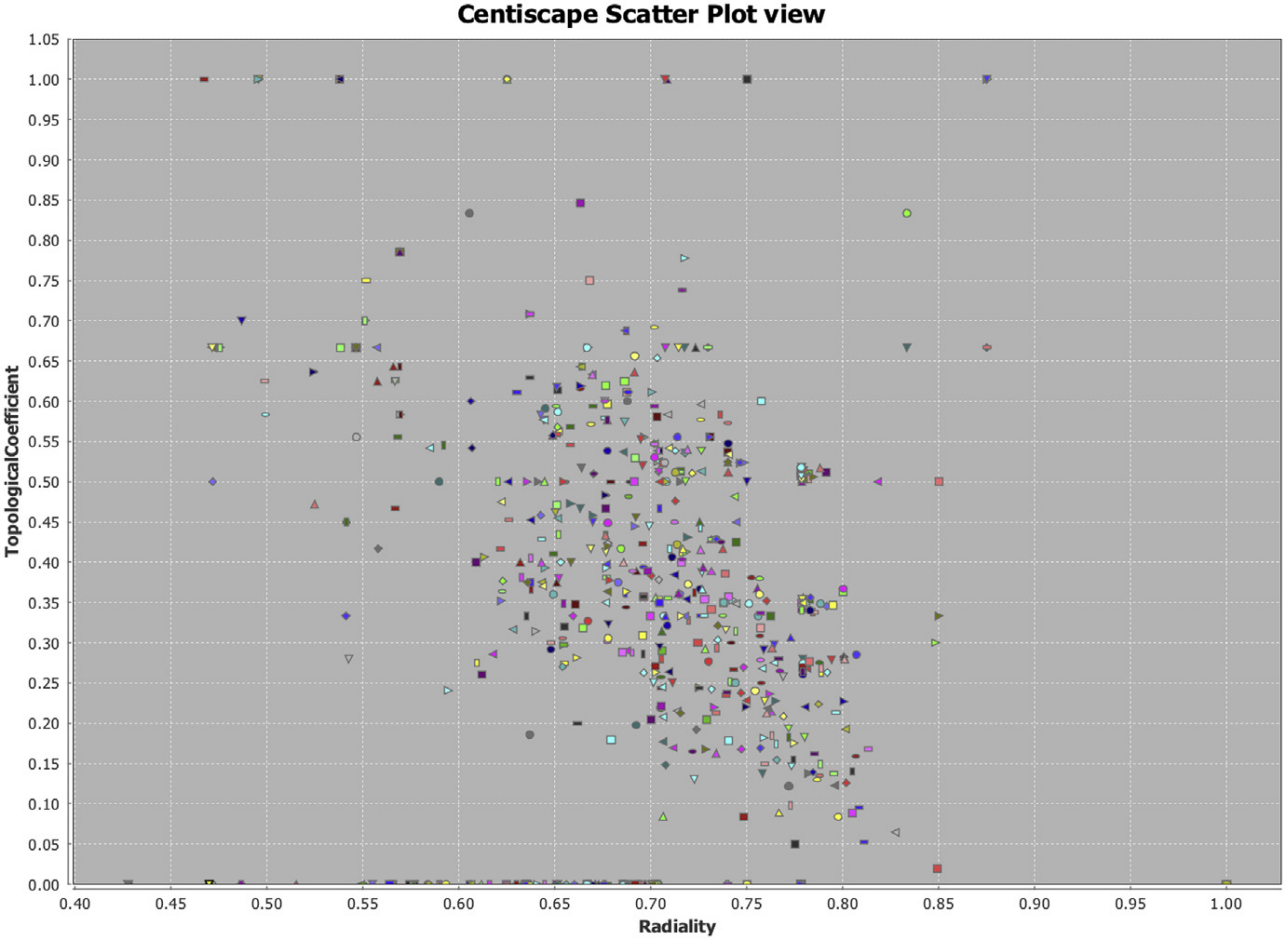
Topological Coefficient (Tn) vs Radiality. Exemplification of Tn as a more suitable measurement for hubs detection using radiality as discrimination point. Since nodes that reaches the closest values to 0 or 1, can be discriminated to latelily bee correlated to its specific interpretation.

**Fig. 9:**
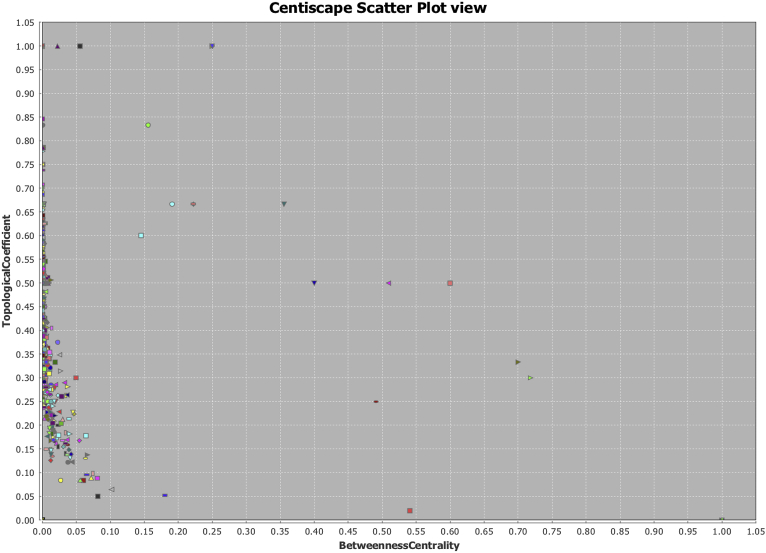
Topological Coefficient (Tn) vs Betweenness Centrality. Using Betweenness as discriminator central measurement, we can observe that nodes then to cluster at origin and disperse over the vertical axis, making more difficult a correct discrimination of hubs.

**Fig. 10:**
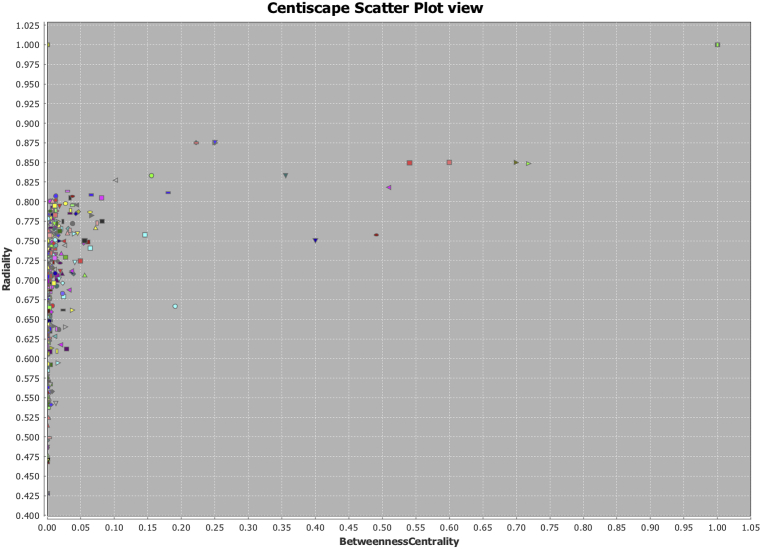
Radiality vs Betweenness Centrality. In this case clustering between two central tendency measurements is denser, this shows that Tn is a better fit to perform a comprehensive node search, allowing a more disperse distribution.

**Fig. 11:**
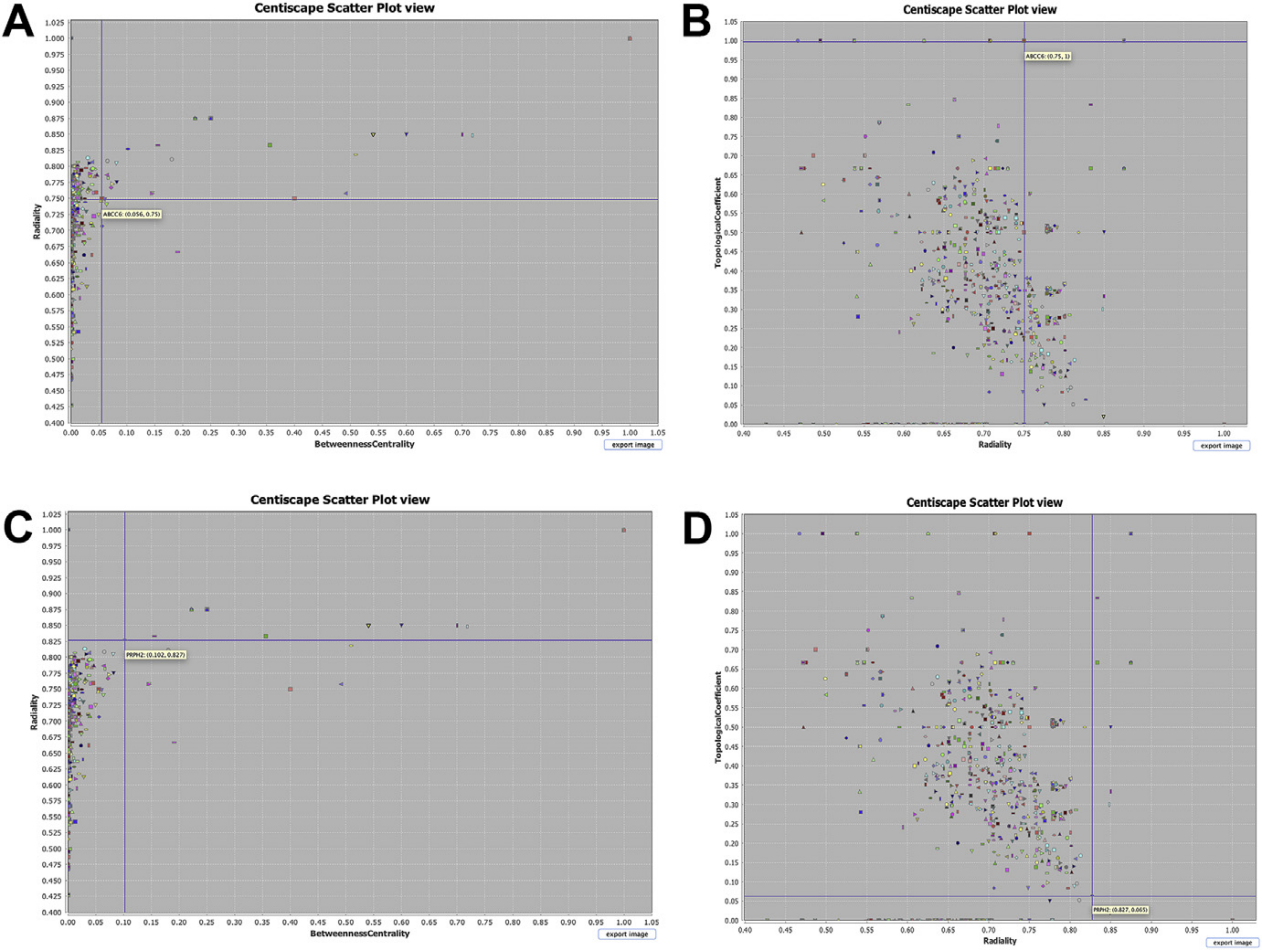
A). ABCC6 Gene in Radiality vs Betweenness plot. B) ABCC6 Gene in Tn vs Radiality plot C) PRPH2 Gene in Radiality vs Betweenness plot. D) PRPH2 Gene in Tn vs Radiality plot. Here we can observe two genes in direct comparison, between Tn and central measurements. PRPH2 can be identified in Tn and betweenness plots. However, ABCC6 gene (Hub), could not be identified using Betweenness based on high clustering, showing efficacy of Tn for hub.

After examining individual results of Topological coefficient, it was calculated Radiality and Betweenness to discern importance of the hubs in the network on topological representation. Three graphs were generated: Tn vs Radiality (Fig. 8), Tn vs Betweenness (Fig. 9), and Radiality vs Betweenness (Fig. 10), to exemplify how using only the topological structure as primary parameter **Tn** is a more suitable measure to find relevant nodes (hubs) in a complex network. Once the networks were analyzed reconstructed and weighted for Tn and centralities, the top ten markers using radiality as order factor were selected from Genes Network and Protein Network obtaining ranges of Tn and Betweenness from 0 to 1 spectrum (Table 3). In both networks a total of 6 genes were repeated amongst the top ten, two of them (ABCC6 and PRPH2) were selected (Highest and lowest Tn) to demonstrate differences between both measures on detection (Fig. 10). When comparing Tn and Betweenness on Gene ABCC6 (Fig. 11A and B) ABCC6 shows a clearer signal on Tn graph, that is difficult to pick up using the centrality measure, for gene PRPH2 (Fig. 11C and D), the signal is clear enough on both datasets to be discriminated by any measure.

**Table 3:** Comparison between topological coefficient (Tn) and betwennes (Centrality measure), using Radiality as discriminator factor.

Network	Gene/Protein	Tn	Radiality	Betweennes
Disease to Genes Network	PGK1	0,0000	1,0000	0,0000
	CLN7	0,6667	0,8750	0,2222
	MFSD8	0,6667	0,8750	0,2222
	TREX1	0,5000	0,8500	0,6000
	PRPH2	0,0647	0,8272	0,1018
	CMT6	0,5000	0,8182	0,5091
	RPGR	0,0953	0,8085	0,0652
	RHO	0,0882	0,8050	0,0812
	ABCC6	1,0000	0,7500	0,0556
	CLN3	1,0000	0,7083	0,0222
Disease to Proteins Network	P00558	0,0000	1,0000	0,0000
	A0A076V826	0,0781	0,8532	0,1297
	P08100	0,0781	0,8532	0,1297
	P23942	0,0664	0,8061	0,0416
	A0A087WTS9	0,1907	0,7941	0,0041
	H7C4H4	0,1144	0,7926	0,0098
	Q9NZN9	0,3333	0,7888	0,0018
	B1ALA7	1,0000	0,7500	0,0500
	A0A0G2JMG3	1,0000	0,7000	0,0250
	A8JYI8	1,0000	0,6942	0,0000

	**Repetead Genes on Top of Both Networks**			
	6	PGK1	PRPH2	RPGR
		RHO	ABCC6	CLN3

**Fig. 12:**
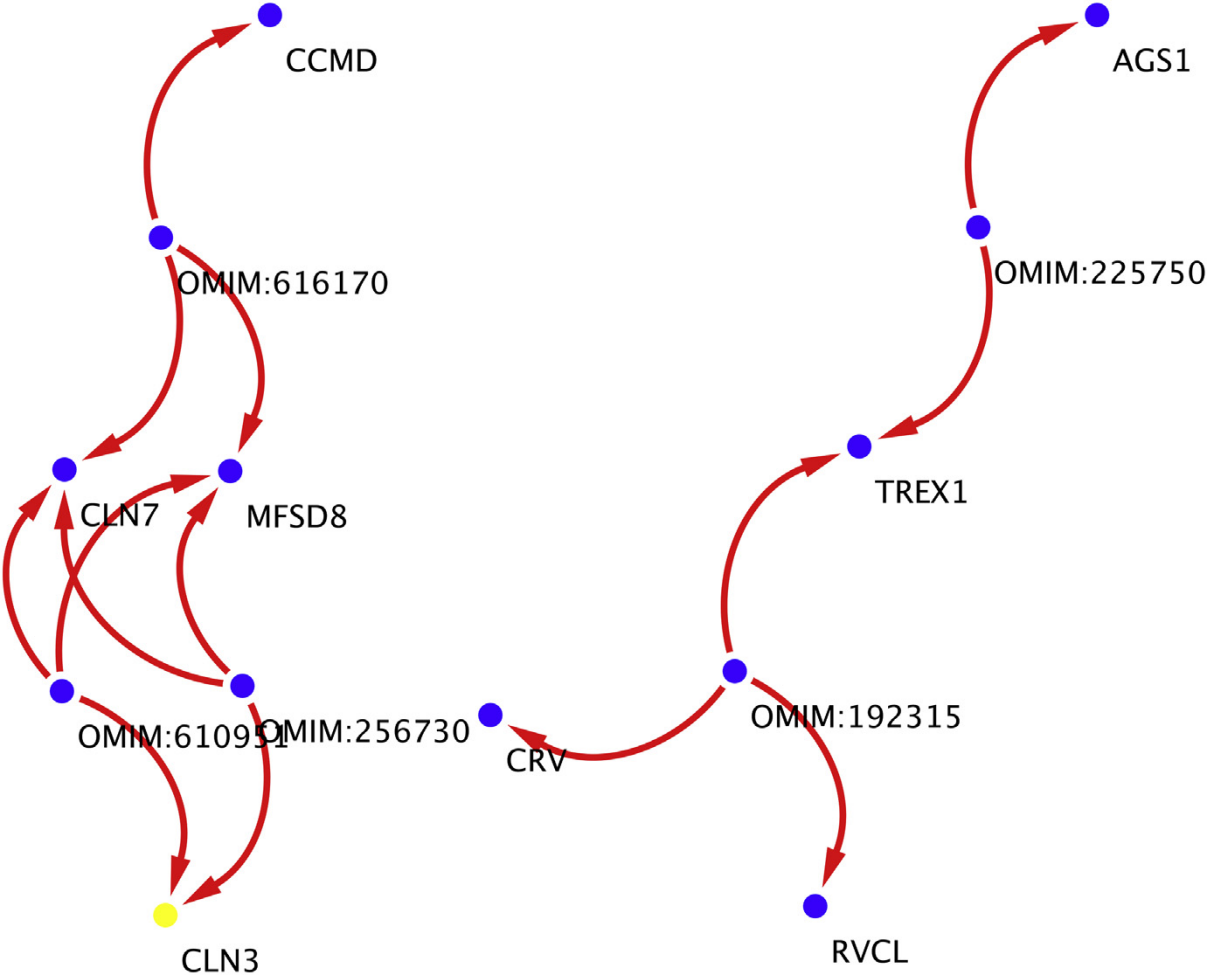
CLN3-CLN7 subnetwork. This subnetwork is formed when performing a disaggregation experiment over CLN3. The CLN3 subnetwork is related to TREX1 subnetworks through CRV gene.

The disaggregation network analysis was performed with the next highest Tn gene on the list (CLN3) and shows that this gene can be map as an independent subnetwork of main network holding 9 vertex connection, and this new subnetwork is ligated to another two genes inside the list (CLN7 and TREX1) Fig. 12.

**Fig. 13:**
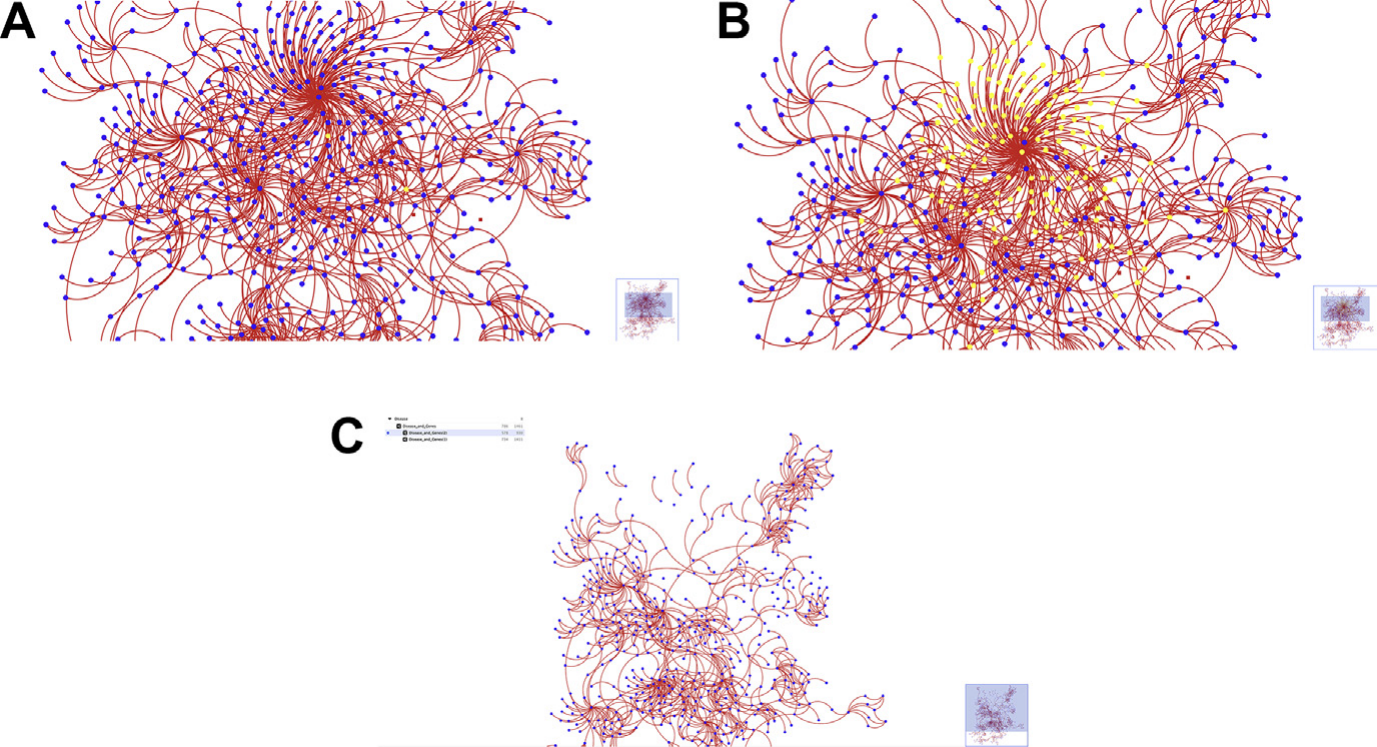
Disaggregation of RHO gene inside Disease to Genes Network A). Selection of RHO gene inside network. B) Expanding selection of RHO-RP5 genes complex to its closest neighbors C) Subtracting all nodes related to RHO gene from the network. This 3-step process shows the importance of RHO gene inside the network, since once RHO gene is selected RP5 gene automatically is selected with it, suggesting and not previously noted relationship. When the RPO-RP5 complex is subtracted a great portion of the network is disassembled, suggesting his importance as a biological hub.

The same disaggregation experiment was performed with the lowest gene on the list (RHO), the primary analysis shows that this gene was centrally positioned and share the same topology characteristics with genes RP5 (not on the list), appearing as two individual nodes but so ligated between them that subtracting one cause the subtraction of the other one (Fig. 13A). After subtraction from network of RHO gene connection network (Fig. 13B), the original Disease Genes Network that contains 786 nodes and 1461 edges previously connected, diminish to 578 nodes and 930 edges and multiple isolated nodes (Fig. 13C).

## 4 Discussion

One remarkable finding is the increased coverage provided by this network, relative to previously available databases information, as seen in the gene symbol PRPH2. In this specific scenario, PRPH2 has 21 diseases linked to one hub (Fig. 2). When searching for the same gene in the RPGeNet database, the result has a node degree of only two as shown in Fig. 2. This is one example showing the differences in the recently created RetinalDiseaseNetwork from the previously existing RPGeNet.

**Fig. 14:**
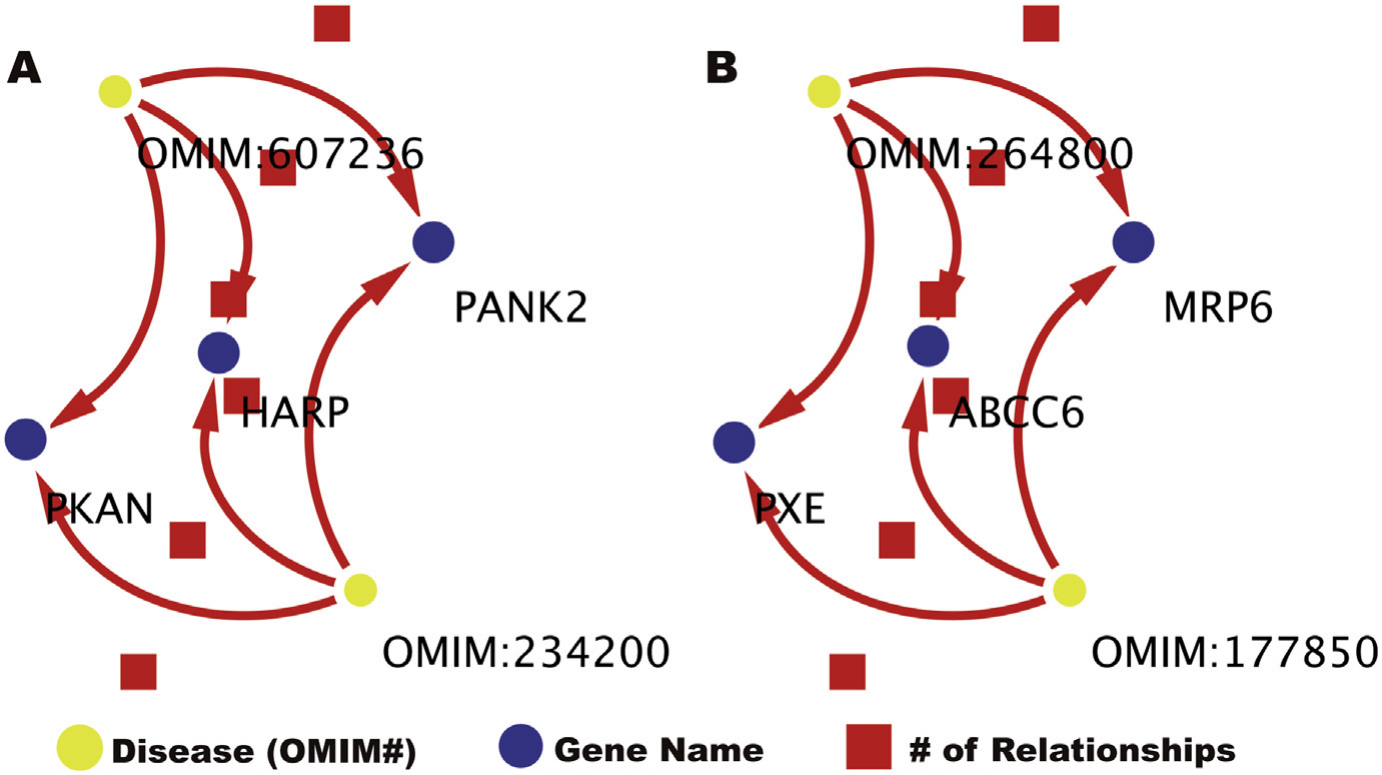
Network view comparison of two sets of diseases in close network proximity. Diseases of OMIM# 607236 to 234200 in Fig. 2A and 264800 to 177850 in Fig. 2B are equidistant in the network.

Another result from this work is the observation that proximity within the network can be attributed to a multigenic diseases or that diseases share the same genotypical pathway as shown in Table 1, where the similarity corresponds to a Forte form of the same disease, but this scenario is not necessarily true fall all related cases. As shown in the comparison of two sets of diseases (OMIM# 607236 to 234200 and 264800 to 177850) where unrelated diseases are equally proximal to each other (Fig. 14A). compared to related diseases that share the same genetic background (Fig. 14B) [12]. This is interesting because Fig. 14A relates diseases that may have not been related previously, but now it is shown an association between two very different diseases.

The disease-gene network had the most accurate power law model with a correlation = 0.814, r-squared = 0.810, that power law correlation means the strong presence of hubs. However, the disease phenotype network with correlation = 0.990, r-squared = 0.788, despite a lower r-squared proves to have the best fit into hubs clustering as shown by Fig. 4A.

For the protein-disease network there appeared to be at least some of hubs present, based on the correlation = 0.556, r-squared = 0.654, and a good linear fit in Fig. 6A, but when the TP was analyzed (Fig. 6B), it shows a high dispersion of nodes and clustering bellows the fit, stating an unlike possibility of the presence of hubs of biological significance in this network.

Node degree distributions for disease-gene with common phenotype and disease-phenotype with common gene are not as strongly correlated to the power law model (Fig. 7A). Regardless of this poor correlation the TP fit, shows the presence of clustering areas but with several neighbor inferior to 10, making worth it a posterior analysis on this area to determinate if clustering is related to multiple variant forms of the same disease.

In all the node degree distributions there are some outliers that are affecting the fitted line and correlation valued. To highlight the effect, this leads to review the disease-gene network. This node degree distribution had a high correlation, with only one outlier. With the removal of the one outlier, OMIM:268000, when revisit the node degree distribution in Fig. 4. It is seen that the r-squared value goes from 0.810 to 0.895 with the removal of just one outlier.

After the analysis of those multiple networks, the TP distribution and correlations allows to find many hubs, that must be individualized and studied according its clinical and biological significance.

For this reason, was necessary to conduct a second layer of experiments to evaluate the importance of the detected hubs on the four networks. As described in the methods section Radiality, Betweenness and Topological Coefficient was determined for the four main networks, however only two provide a clean layer of information that can be mapped to genes and therefore performed a disaggregation experiment. As shown on Table 3, the Radiality measure in conjunction with Tn can be used to discern in a proper way node (hubs of biological importance). Being values of Tn closer to 1 index of subnetworks inside the primary networks and values closer to 0, will indicate the presence of clustering nodes (hubs) that holds primary functions ass holders of network integrity, in biological terms nodes that are necessary to aim, and perform functional analysis.

Even though the whole gamma of experiments were conducted, and multiple central nodes were detected, the network analysis did not show any superhub, that could have been an inflection point for pursuing a clinically important connection between genes and related diseases. This could be due to the limitations of this study, that includes the number of diseases present. in big data mining and network informatics the bigger the dataset the more powerful the interrelationships that can be found, so increasing the number of ontologies will improve the nodes detection system.

Another limitation found in this study is the difficulty of compare the results with other dataset beyond specific examples, since those datasets do not incorporate disease-phenotypic information (except OMIM) and uses global (path-related = shortest-pathway) algorithms (e.g PRGeNet), that when analyzed against the use of Tn distributions could not be properly compared.

Finally, this new database can be used to find a variety of information besides hubs, using this approach; we were able to find the ten most common phenotypes occurring in diseases and get number of occurrences for those phenotypes and also find more than 10 genes (Table 3) that can be targeted for posterior biological analysis [1, 12].

This shown the efficacy of a comprehensive biological database on retinal diseases, that sets the bases for posterior works on the networks provided. Where the evidenced hubs should be studied in greater depth, and the genes proposed by this paper can be used for a targeted sequencing posterior analysis on a prospective cohort of patients.

## 5 Conclusions

The presence of hubs in this kind of networks reflects the existence of common pathways in gene expression in different and unrelated diseases; however, sharing the same gene could not involve sharing the same phenotypic results or evolution on the disease.

This database could be used to find the most common phenotypes related to genotypes, allowing to explain complicated paths on how co-expression on multiples genes lead to a specific phenotype.

Hubs with scale-free networking were identified to be present strongly in the disease-gene network, and somewhat strong in the protein-disease network. Where found unexpected actors such the gene PRPH2 that appears in at least 21 diseases connected by at least one node of distance, and in other multiple diseases at two or more nodes, or subnetworks as represented by gene CLN3 interacting with TREX1.

It is difficult to explain the complexity of all nodes, their connections and interactions. However, currently this tool could provide a bunch of grouped targets, for performing a gene hunt that can unveil relationships between actors (diseases, phenotypes, genes, proteins, etc.) that cannot be perceived other way.

In this work we only explored four networks with interconnection across multiple genes, phenotypes and proteins that are expressed in multiple diseases. Nevertheless, adding more layers of complexity (e.g methylation patterns, pharmacogenomics) to this networks, based on the analysis of free-scale nodes, could reveal the existence of more hubs (nodes) over all networks (genes, diseases, proteins) that serves as point of interest to expand the understanding on how diseases that are consider one entity, could be related in a closer way or could be more distant apart that the current understanding of network interactions shows.

## Declarations

### Author contribution statement

José-Miguel Lázaro Guevara: Conceived and designed the experiments; Performed the experiments; Contributed reagents, materials, analysis tools or data; Wrote the paper.

Bryan Josue Flores-Robles: Conceived and designed the experiments; Analyzed and interpreted the data; Contributed reagents, materials, analysis tools or data; Wrote the paper.

Karen Garrido, Valvanera Pinillos-Aransay, Leticia Merino Meléndez, Angel Elena Ibáñez, Juan-Antonio López-Martín, Raquel Victoriano Lacalle: Contributed reagents, materials, analysis tools or data.

### Funding statement

This research did not receive any specific grant from funding agencies in the public, commercial, or not-for-profit sectors.

### Competing interest statement

The authors declare no conflict of interest.

### Additional information

No additional information is available for this paper.
